# New-Onset Bullous Pemphigoid in a COVID-19 Patient

**DOI:** 10.1155/2021/5575111

**Published:** 2021-06-07

**Authors:** Natalie Olson, David Eckhardt, Angela Delano

**Affiliations:** ^1^Rocky Vista University, Parker, CO 80134, USA; ^2^St. Anthony North Family Medicine Residency Program, Denver, CO 80208, USA

## Abstract

This manuscript presents a report of bullous pemphigoid rash associated with COVID-19 for the first time. The objective of this manuscript is to present a unique dermatological case in the setting of a COVID-19-positive infection to further recognize the virus symptomatology. A 37-year-old female with a past medical history of class III obesity, type II diabetes mellitus, and hypertension presented to the emergency department in September 2020 with inpatient and outpatient follow-up through to November 2020. The patient denied any personal or family history of skin disorders. The patient tested positive for COVID-19 prior to hospitalization and presented to the hospital with severe, persistent, pruritic rash meeting dermatopathological, serologic, and clinical criteria for bullous pemphigoid diagnosis. Histopathology H&E punch biopsy from her left flexor wrist demonstrated epidermal keratinocyte necrosis, subepidermal vesiculation with eosinophils, gossamer stranding of the papillary dermis, and subepidermal edema. Direct immunofluorescence punch biopsy from her left flexor wrist demonstrated strong linear IgG staining at the dermoepidermal junction, with weaker and focal linear C3 staining. Antigen-specific serology was consistent with bullous pemphigoid. There was no previously reported cutaneous association of COVID-19 infection with bullous pemphigoid making this case an important addition to the body of evidence helping to identify bullous pemphigoid in the setting of viral infection.

## 1. Introduction

In 2019, a (Novel) coronavirus emerged in Wuhan, China. The virus has been found to cause severe pneumonia and has affected 35 million people, resulting in 1.5 million deaths worldwide since its emergence. Severe acute respiratory syndrome coronavirus 2 (SARS-CoV-2) is a single-stranded RNA virus that spreads via aerosolized droplets and enters host cells through the angiotensin-converting enzyme 2 (ACE2) receptor [1]. It is widely accepted that high ACE2 expression has been found in the type II alveolar cells and secretory cells of the lungs; however, it has also been suggested that ACE2 receptors are found in the small intestinal epithelium, oral epithelial cells, endothelial cells, and keratinocytes in the skin [1]. Though most symptomatic patients who contract the virus present with flu-like symptoms including fever, cough, fatigue, and malaise, recent evidence suggests that some patients have also experienced cutaneous manifestations of the disease. It is important to consider the dermatologic manifestations of SARS-CoV-2 as they may help to guide practitioners towards the diagnosis of COVID-19 among other respiratory viruses. Here, we present a report on a unique dermatological presentation of bullous pemphigoid (BP) in a known COVID-19-positive patient.

## 2. Case Report

Our patient is a 37-year-old female with a past medical history of type II diabetes mellitus, hypertension, and class III obesity who presented to the emergency department in September 2020 with a worsening rash. On the day of admission, she noted the development of red, raised lesions on her arms over the course of the last week. The lesions were mildly pruritic and nontender. The following day, she began experiencing typical COVID-19 symptoms (fever, shortness of breath, and muscle aches) and tested positive for COVID-19 on day 3 of her symptoms. She had no personal or family history of dermatologic or autoimmune conditions. No recent changes in the environment, detergent, soaps, or lotions were reported. No recent immunizations or changes in medications were reported. Medications included lisinopril 20 mg QD since February 2020 for hypertension and metformin 500 mg BID since February 2020 for type II diabetes mellitus.

The rash spread to her lower extremities, torso, and ultimately onto her neck and chin over the ten days before she presented to the ED. The lesions became larger, and some started to blister. The severity of the pruritus and discomfort motivated her to come to the emergency department. She was found to be tachypneic but oxygenated well on room air. Supplemental oxygen was given at 1-2 L by using a nasal cannula to relieve tachypnea with no hypoxia noted during hospitalization.

**Figure 1:**
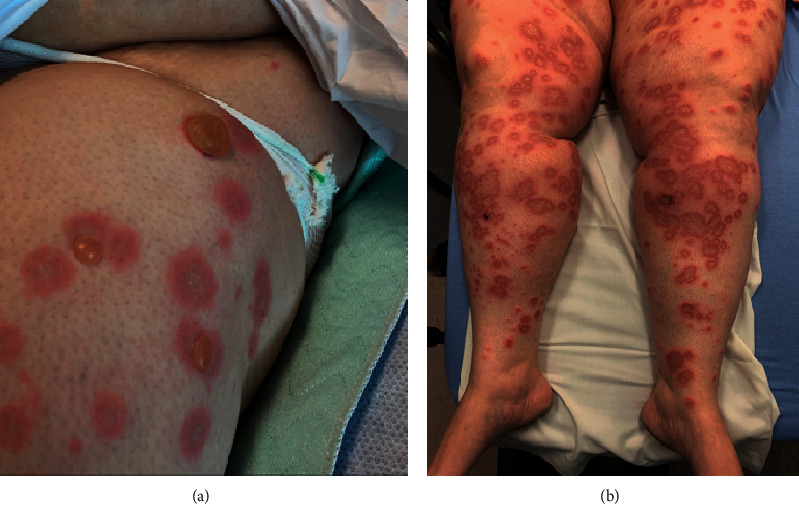
(a) Left hip with blanching and tense blisters that are tender to palpation. (b) Bilateral lower extremities with erythematous, scaly annular lesions which are pruritic and at varying stages of the disease.

On examination, the patient reported negative Nikolsky sign, blanching, blisters tense and tender to palpation, and scaly lesions nontender to palpation. No mucosal involvement was observed on physical exam (Figure 1).

**Figure 2:**
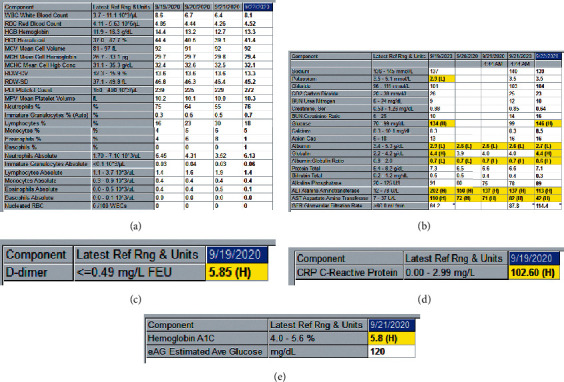
Laboratory results. (a) Complete blood count. (b) Complete metabolic panel. (c) D-dimer. (d) C-reactive protein. (e) Hemoglobin A1C.

Hematologic studies for the patient were found to be normal. On admission, she did demonstrate mild hypokalemia (which resolved during her hospital stay), elevated blood glucose (serum ranged from 134 mg/dL to 146 mg/dL and HbA1C 5.8%), hypoalbuminemia, and elevated liver enzymes (ALT and AST). A D-dimer and C-reactive protein were also ordered and found to be elevated. Figures 2(a)–2(e) demonstrate the laboratory results for the patient at the time of admission, including abnormal values highlighted in yellow.

Table 1 lists the results of skin biopsy, direct immunofluorescence, and antigen-specific serologic testing for the patient. Pathology images were not available.

Differential diagnoses were erythema multiforme, chemical burn or exposure, leukocytoclastic vasculitis, drug reaction, granulomatosis with polyangiitis, urticarial vasculitis, and other viral exanthems.

She was treated with oral diphenhydramine, topical diphenhydramine, triamcinolone 0.5% cream, and IV dexamethasone 6 mg. Her home medications (metformin and lisinopril) were held during her hospital stay, and the patient was placed on sliding-scale correctional insulin for persistently elevated blood glucose values. Lisinopril was withheld due to the association with bullous pemphigoid, though the patient had been taking it without issues for years. She was discharged on oral dexamethasone 6 mg to complete a 10-day course in addition to topical diphenhydramine and triamcinolone. Since discharge, she has repeated multiple steroid courses due to the persistent discomfort associated with the rash as it improves with steroids and then flares back up upon completion of steroid courses. She has also been taking nicotinamide to help with her rash. Her dyspnea, body aches, and fever resolved shortly after discharge, and the rash was her only remaining symptom.

The patient has completed multiple steroid courses with continued exacerbations of the rash. At the end of November 2020, she was starting her third prednisone taper since discharge in addition to doxycycline. At the time of publication, immune-modulating therapies were under consideration but not yet initiated.

Pathological disease course since initial admission is as follows:

- First clinic visit on 11/17/2020: the patient was started on doxycycline 100 mg BID and nicotinamide 500 mg TID, and prednisone taper was initiated. She initially declined immunosuppressive agents given the COVID-19 pandemic.
- Telehealth visit on 12/9/2020: the patient stopped doxycycline and nicotinamide as per the provider's recommendation.
- The patient was admitted on 12/11–12/14 for bullous pemphigoid flare requiring hospitalization for pain control and wound care.
- Office visit on 12/29/2020: the patient noted worsening with the painful blister formation on the face, arms, groin, legs, and back, with interference with the ability to sleep and work.
- The patient received two rituximab infusions on 1/11/2021 and 1/25/2021. She continued hydroxyzine 25–50 mg nightly QHS for pruritus.
- Pt was seen at dermatology clinic on 3/23 and is doing very well after the second rituximab infusion. Has no blisters and is down to 7.5 mg prednisone. Will continue to slowly decrease dose. Patient to follow up in 4 weeks.

Verbal consent for the use of images and the case report was obtained from the patient. No written consent has been obtained from the patient as there are no identifiable data included in this case report.

## 3. Discussion

This submission presents a review of the current literature related to cutaneous manifestations of SARS-CoV-2 and the case of a woman who tested positive for COVID-19 after she noticed an annular, bullous, pruritic rash on her extremities two days prior to her diagnosis. The clinical picture of her rash was consistent with bullous pemphigoid, a condition which, to date, has not been associated with COVID-19.

In a recent literature review, Gisondi et al. reported on novel cutaneous manifestations of COVID-19 and discovered several dermatological presentations. These included exanthems (varicella-like, papulovesicular, and morbilliform rash), vascular (chilblain-like, purpuric/petechial, and livedoid lesions), urticarial, and acropapular eruption [2]. Our patient, who presented with a bullous, pruritic rash, does not quite fit into any of these reported categories, making her case especially unique as there are no published data on bullous dermatological patterns in COVID-19 patients to date. Perhaps, the most similar reported presentation is the varicella-like manifestation, which was recently found to be a possible early and quite specific manifestation of COVID-19 [2].

A popular opinion among cutaneous involvement of COVID-19 is that patients experience varying types of vasculitis, one being its effect on the dermal/epidermal junction of the skin. Becker examined the effects of COVID-19 on the circulatory system and found multiple case reports in which urticarial vasculitis was implicated [3]. Upon histopathologic examination of biopsy-derived material, dermatitis and vascular degeneration of the basal epidermal layer were evident. Endotheliitis within lymphocytic infiltration of the dermal vesicles and arterioles and microthrombosis of papillary dermal capillaries are also common findings [3].

There have also been numerous case studies that have described dermatologic conditions associated with COVID-19, which do not necessarily fall under the category of vasculitis. Due to the difficulty in accessing dermatology consultation during the pandemic, skin manifestations of the disease may be underrecognized, and it is important to study them further.

While COVID-19-related dermatologic conditions have been relatively well documented in the literature, our patient's presentation of bullous pemphigoid has not previously been associated with COVID-19 infection. Bullous pemphigoid is an uncommon intraepidermal blistering disease caused by autoantibodies to adhesion molecules expressed in the skin and mucous membranes. The bullae appear spontaneously and are painful when they rupture. Bullous pemphigoid is a relatively benign pruritic disease characterized by tense blisters most commonly seen in flexural areas, with a course characterized by exacerbations and remissions [4]. The disease most often presents in patients who are 40–60 years old and with prodromal symptoms including pruritus, dermatitis, or urticaria. Lesions may occur anywhere on the skin surface in a localized or diffuse manner but are most frequently found on the flexural surfaces of the arms, axillae, legs, groin, and lower abdomen [5]. Our patient's presentation seems to fit this clinical picture with multiple exacerbations of her rash after initial treatment and the location of her rash being on the arms, groin, legs, and back.

Bullous pemphigoid flares are associated with the binding of autoantibodies to the proteins of hemidesmosomes triggering an inflammatory pathophysiological process ultimately leading to dermal-epidermal junction cleavage and blister formation [6]. It is thought that both inflammatory infiltrating cells and skin-resident cells, including keratinocytes, fibroblasts, and endothelial cells, produce cytokines and chemokines which contribute to the pathophysiologic process of bullous pemphigoid [6]. Interestingly, in many patients who have suffered severe cases of COVID-19, one of the main causes of multiorgan failure is thought to be related to a cytokine storm in which excessive amounts of inflammatory cells lead to organ damage in the lungs and liver. According to an article published in the *Mediators of Inflammation* journal, cytokines associated with COVID-19 included IL-1B, IL-17, and TNF-*α*, which have also been implicated in patients with bullous pemphigoid [6]. This finding could be related to some form of cytokine crossover in patients who present with cutaneous manifestations of the disease and should be studied further to gain a deeper understanding of the mechanism related to the inflammatory response of COVID-19. Lab values for our patient demonstrated an elevation in alanine aminotransferase (ALT) and aspartate aminotransferase (AST), which may indicate damage to the liver, as well as elevations in C-reactive protein, a marker for acute inflammation. Interleukin values were not measured at the time of admission, and as the patient was afebrile throughout her hospital stay, we did not believe that the patient was experiencing the systemic inflammation related to cytokine storm seen in some patients with COVID-19.

It is well understood that the underlying cause of bullous pemphigoid is due to autoantibodies that are directed at adherence proteins within the skin; however, little evidence exists that discusses the triggering factors for the development of such autoantibodies [7]. Patel et al. proposed that the etiology of bullous pemphigoid is likely multifactorial in nature, with components of environmental triggers, genetic predisposition, and a potential role of complement deposition due to evidence of complement seen within the blister fluid [7].

There have been case reports of patients developing bullous pemphigoid following vaccine administration and viral infection [8]. A popular theory is that introduction of a vaccine or virus leads to cellular activation of IL-17 and ultimately the release of proinflammatory cytokines and proteolytic enzymes, which may result in disruption to hemidesmosomes and blister formation [8].

Though recent literature reports a strong correlation between viral infections and the development of BP, it is prudent to consider that most patients diagnosed with pemphigus rashes have a genetic predisposition to the development of autoantibodies directed towards the dermal proteins BP180 and BP230, which play a critical role in maintaining epidermal adhesion [7, 9]. It is important to recognize that individuals with a genetic predisposition who are exposed to certain medications may develop drug-induced BP, including DPV inhibitors such as vildagliptin and linagliptin and certain diuretic medications such as furosemide and spironolactone, as well as certain antipsychotic medications and checkpoint inhibitors [10]. The second class of drugs that has been associated with bullous pemphigoid is angiotensin-converting enzyme inhibitors, or ACE-Is, specifically losartan, valsartan, and lisinopril [11]. Interestingly, our patient had been taking lisinopril. Although a pemphigus reaction to lisinopril is rare, this medication was stopped upon the patient's first admission. It is unlikely that her symptoms were secondary to her medications as the symptoms usually resolve upon discontinuing the medication, which is not the case here. It is also less likely as the patient had been taking the medication without issues for nearly 20 years.

Recent studies have reported that nearly all patients who develop pemphigoid rash have an increased frequency of the DQB1*∗*0301 allele with some studies showing that the BP180-specific Th1 and Th2 cells are restricted to HLA-DQB1*∗*0301 [7, 9, 12–14]. Of note, the BP180 and BP230 antibodies were detected in our patient. Though we did not perform genetic testing on our patient, it is important to recognize that her genome could have contributed to the development of her rash.

The role of complement deposits found within the blister fluid is also an important consideration in the etiology of BP, especially in our patient who was newly diagnosed with COVID-19. Romeijn et al. cited that complement deposits are found in the blister fluid of 83.1% of the skin biopsies of patients diagnosed with bullous pemphigoid [15]. This is important to consider based on Java et al.'s report on the coronavirus ability to activate complement pathways and their potential involvement in the severity of the disease [16]. It would be interesting to understand more thoroughly the involvement of complement pathways in both BP and COVID-19 and their correlation with one another.

It is important to note that it is entirely possible that our patient had developed both conditions simultaneously without one precipitating the other. It would be prudent to consider the possibility that the two disease processes were unrelated conditions in this patient. Further investigation for a causative relationship between COVID-19 and BP is warranted.

## 4. Conclusion

This case presents a unique dermatologic presentation of COVID-19 and recent literature on potential pathophysiology of BP in COVID-19-positive patients. SARS-CoV-2 enters the host cell through the ACE2 receptor, which is found on multiple cell types including dermatological cells. Once it enters into a host cell, the virus activates complement, and, in turn, inflammatory cells are recruited to the cell eliciting a response [16]. There has been speculation as to the contributing etiology of bullous pemphigoid, and this case brings to light new evidence of COVID-19 as a potential triggering event in patients with BP and should be studied further.

This research is important for medical practitioners to continue to understand COVID-19 and associated dermatological manifestations. The pathogenesis of both COVID-19 and bullous pemphigoid is complex. Perhaps, their relation to one another as in this case will allow for a deeper understanding of each disease and the mechanisms by which they evolve and progress.

### 4.1. Patient Perspective

“It's been really horrible and difficult. The itching and the pain have been difficult to deal with. The outcome of having bullous pemphigoid so young makes me worried. It's supposed to take years to fully go away and usually happens to older people. It's embarrassing when the blisters are on my arms and people can see them. If it is from COVID, then people should really listen when they tell us not to attend group gatherings.

I experience worsening symptoms whenever I take less than 30 mg of prednisone.

It's difficult to sleep at night because the blisters on my back are so itchy and it's hard to lay on them. Insurance only pays for a small amount of topical creams and I need to use so much over my whole body, so it doesn't really work as an option. Hot showers sometimes help the itching, but I still don't sleep well. It's just so difficult and I hope it gets better.”

## Figures and Tables

**Table 1:** Patient lab results.

Diagnostic	Location/type	Result	Normal value
Biopsy	Left flexor wrist	Epidermal keratinocyte necrosis, subepidermal vesiculation with eosinophils, gossamer stranding of the papillary dermis, subepidermal edema	<20 RU/mL (negative)
Direct immunofluorescence	Left flexor wrist	Strong linear IgG staining at the dermoepidermal junction, with weaker and focal linear C3 staining	≥20 RU/mL (positive)
Antigen-specific serologic testing	Bullous pemphigoid 180 IgG Abs	211.4 RU/mL	<20 RU/mL (negative)
	Bullous pemphigoid 230 IgG Abs	<1.0 RU/mL	≥20 RU/mL (positive)

## Data Availability

The case report, meta-analysis, and scientific data used to support the findings of this study are included within this study. These prior studies are cited at relevant places within the text as references [1–15].
